# Comparing the Efficacy of Landmark-Based Fascia Iliaca Compartment Block and Pericapsular Nerve Group Block for Preoperative Positioning and Postoperative Analgesia in Patients Undergoing Surgery for Hip Fractures: A Randomized Controlled Trial

**DOI:** 10.7759/cureus.67196

**Published:** 2024-08-19

**Authors:** Balachandran Pavithra, Ramamurthy Balaji, Dheepak Kumaran, Balasubramaniam Gayathri

**Affiliations:** 1 Anaesthesiology, SRM Medical College Hospital and Research Centre, Chennai, IND

**Keywords:** elderly trauma, acute pain, postoperative analgesia, spinal positioning, hip fractures, nerve blocks

## Abstract

Background: Subarachnoid block is the most common anesthetic technique for patients having corrective hip surgeries. However, adequate positioning for a successful subarachnoid block is a major challenge in this particular population of patients, owing to the site of fracture. Regional anesthesia, in the form of nerve blocks, is an effective means of alleviating such constraints and gives an added benefit of prolonged postoperative analgesia. The pericapsular nerve group (PENG) block and the fascia iliaca compartment block (FICB), under ultrasonography guidance, are a few examples of the commonly performed peripheral nerve blocks in such settings. However, the landmark-based techniques of nerve blockade still hold good in many resource poor settings, given the lack of ultrasonography facilities.

Aim: To compare the ease of spinal positioning using the patient sitting satisfaction score between the landmark-guided FICB and PENG block.

Materials and methods: This study was done on 80 patients of the American Society of Anesthesiology (ASA) grade I or II with intertrochanteric fractures of the hip joint scheduled for proximal femoral nailing. Patients were allocated into two groups of 40 each through computer generated random numbers, to receive 30 ml of 0.5% ropivacaine via the landmark-guided technique of FICB in Group F and peripheral nerve stimulator assisted landmark-guided PENG block in Group P, 30 minutes prior to spinal positioning. Time to passive leg raise (PLR) to 15 degrees and time to PLR to 30 degrees with a standard goniometer guidance at visual analogue scale (VAS) score < 4 and ease of spinal positioning through the sitting satisfaction score at the 30th minute were assessed. Any adverse effects and events of failure were noted. The duration of postoperative analgesia was measured through the time to the first dose of paracetamol on arrival at the post-anesthesia care unit.

Results: Statistical analysis was done using JASP version (0.18.3.0) using the independent samples t-test and significance was taken when p value was < 0.001. The time to PLR to 15 degrees and 30 degrees were achieved faster in the patients who received the PENG block in comparison to the patients who received the FICB, and the average patient sitting satisfaction score was significantly higher in Group P as compared to Group F (p < 0.001). While the overall amount of analgesics used in both groups was similar, the overall period of postoperative analgesia was prolonged in Group F compared to Group P (p < 0.001).

Conclusion: The landmark guided PENG block is feasible and superior to the landmark-guided FICB for preoperative positioning and analgesia. The FICB provides a longer duration of postoperative analgesia for patients with intertrochanteric fractures.

## Introduction

The success of subarachnoid blocks is dependent on correct positioning, which can be compromised in patients with fractures of the hip joint primarily due to pain [1]. Lower limb blocks like the femoral nerve block , the fascia iliaca compartment block (FICB), and the three-in-one femoral nerve block have all been practiced across the globe and have provided numerous benefits for positioning, post-operative analgesia, and reduced opioid consumption [2]. The additional advantages of peripheral nerve blocks include early mobilization of patients, reduced postoperative cognitive dysfunction, and reduced periods of hospital stay [3]. The pericapsular nerve group (PENG) block is an addition to this vast armamentarium of blocks and has the added advantage of motor sparing as compared to the other lower limb blocks [4]. These procedures can be performed bedside using the classically described landmark techniques or with ultrasonography guidance. Ultrasonography guided nerve blocks, though more accurate, pose a unique challenge in countries like India, where there is a huge challenge of resource limitation, especially in the outskirts and rural regions, and a lack of adequate training in such ultrasound guided procedures [5,6]. Hence, the gold standard landmark-based techniques still hold good in trauma and orthopedic surgeries.

The landmark-guided FICB and the landmark-guided PENG block are assessed in terms of their efficacy for preoperative positioning and postoperative analgesia in patients with intertrochanteric fractures of the hip joint in this study.

## Materials and methods

This double-blinded, prospective trial was registered at the clinical trial registry of India (CTRI) (CTRI/2023/01/049295). After obtaining documented consent, patients scheduled for proximal femoral nailing of intertrochanteric fractures at SRM Medical College and Hospital, Kattankulathur, were enrolled in this study. Patients between the ages of 20 and 70 years, belonging to both sexes, assessed under American Society of Anesthesiologists (ASA) class I and II, who weighed more than 50 kg, were included in this trial. Participants with a history of severe cardiac disease, chronic kidney disease, chronic liver disease, and respiratory disease, patients with coagulation abnormalities, a history of allergic reactions to local anesthetics, pregnant women, patients with contraindications for spinal anesthesia, and patients who were not consenting to the block were excluded.

After undergoing through preanesthetic evaluation for surgery, the 80 patients who were enrolled were placed into the two groups: Group F (FICB) and Group P (PENG block) using computer generated random numbers. On the day of surgery 30 minutes before the procedure, the study participants were shifted to the operating room, and the baseline vitals were recorded after placing the standard monitors. Prior to the block administration, the visual analogue scale (VAS) score was the tool of choice to measure the intensity of the pain.

The patients were explained the procedure and reassured. All of these blocks were performed by an expert anesthesiologist with over five years of experience or who is known to have performed over 50 nerve blocks. The side of the limb for surgery was identified and under aseptic precautions, the parts were prepared and draped by the first anesthesiologist. Patients who belonged to Group F were administered the landmark-based FICB (infra-inguinal) [7]. The pubic tubercle and the anterior superior iliac spine were identified and with the help of a sterile ruler, a line was drawn connecting the two points. The medial two-thirds and lateral one-third was noted a point was marked on the line. From here, a point 1 cm below was marked. This was considered the point of entry. Local infiltration was given at this point with 2 ml 2% lignocaine. After two minutes of administering the local anesthetic, a 21-gauge block needle (Contiplex needle, Braun Medical, India) was used to enter the point marked perpendicular to the skin. The needle was advanced till two “pop-offs” were felt, and 30 ml of 0.5% ropivacaine was administered with repeated negative aspiration for blood after every five milliliters of injectate. On positive aspiration for blood, the needle was withdrawn and redirected.

Patients who belonged to Group P were administered the landmark-guided PENG block. A peripheral nerve stimulator was included in the PENG block to avoid unnecessary injury to the femoral nerve [8]. Under sterile aseptic precautions the parts were prepared and draped. The pubic tubercle and the anterior superior iliac spine (ASIS) were identified and using a sterile ruler, a line was drawn between them. A point 5 cm medial to the ASIS was identified and marked [8]. Local infiltration with 2 ml 2% lignocaine. After two minutes, a 21 gauge Contiplex® (B. Braun Medical, India) nerve block needle was inserted perpendicular to this point till bony contact was achieved. The Stimuplex® (HNS12, B. Braun Inc.) peripheral nerve stimulator was attached with 1 mA set current, which was protective against direct contact with femoral nerve. This was confirmed with quadriceps contraction and the needle was withdrawn and redirected laterally to avoid femoral nerve blockade or injury. On reaching bony contact, the needle was withdrawn to 1 mm and 30 ml of 0.5% ropivacaine was administered with multiple aspiration. Excessive medial angulation was avoided and constant palpation of the femoral artery was ensured throughout the whole drug administration. Any event of inadvertent vascular puncture, hematoma formation, local anesthetic toxicity was made note of [8].

After administration of either blocks, the timer was started and passive leg raise (PLR) initially to 15 degree was attempted every three minutes. The VAS score was made a note of. Once the patient was comfortable with a 15 degree raise with a VAS score < 4, the time was noted. PLR to 30 degree was attempted and the time to a comfortable 30 degree raise was noted when VAS score < 4. The vitals were also measured for the 30-minute period, after which the patient was put into a sitting position comfortable for spinal anesthesia and the ease of spinal positioning was measured with the patient sitting satisfaction score: 0 = unsatisfactory, 1 = satisfactory, 2 = good, 3 = excellent [9]. Spinal anesthesia was standardized to both the groups with 15 mg (2.5 ml of 0.5% bupivacaine H without any additive agent).

Pain at 30 minutes after administration of the block for passive leg raising to 15 degrees was to be considered as failure. These patients were to be administered Injection Fentanyl 1 μg/kg of bodyweight intravenously and positioned for spinal anesthesia.

All the patients were shifted to the post-anesthetic care unit (PACU) for monitoring of the effective duration of postoperative analgesia. Once the patient complained of pain (VAS score > 4), Injection Paracetamol at a dose of 15 mg/kg of bodyweight was administered. This time was noted, taking baseline from when the patient was shifted to the PACU. Subsequent doses with a minimum six-hour interval was given as per requirement. If the pain still persisted after the first rescue, Injection Tramadol was given at the rate of 1 mg/kg of body weight. Subsequent doses with a minimum 12-hour interval was given if required by the patient. The total amount of analgesics (both paracetamol and tramadol) consumed by the patient was noted.

All the patients were closely monitored for any complaints such as nausea, vomiting, intra and postoperative hypotension, respiratory depression, motor and sensory deficits beyond 24 hour period, bowel dysfunction, and urinary retention.

Statistical analysis was done using JASP (Version 0.18.3). The independent samples t-test was used to analyze the quantitative data. Significance was taken when the P value was < 0.001.

**Figure 1:**
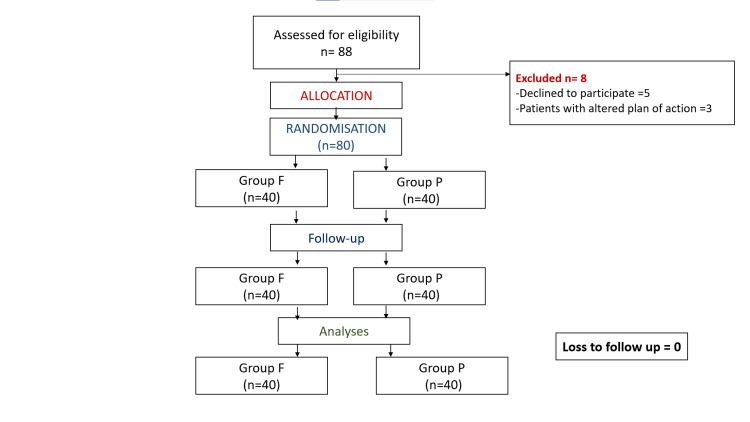
Consolidated standards of reporting trials n: number of patients; Group F: Group receiving FICB; Group P: Group receiving PENG block FICB: Fascia iliaca compartment block; PENG: Pericapsular nerve group

The consolidated standards of reporting trials has been described in the following flow chart (Figure 1).

## Results

In our study, the clinico-demographic data were not statistically significant between the two groups (Table 1). 

**Table 1:** Demographic variation between Group P and Group F Group P: Group receiving PENG block; Group F: Group receiving FICB; n: Number of participants in each group * = p value is insignificant , + = p value is significant. The statistical analysis was done using the independent samples t-test on JASP version 0.18.3. Significance was taken at p < 0.001. PENG: Pericapsular nerve group; FICB: Fascia iliaca compartment block

Demographic Data	Group	n	Mean	SD	Coefficient of Variation	p-value
Age (years)	F	40	57.725	6.102	0.106	0.748*
	P	40	58.150	5.664	0.097	
Height (cm)	F	40	166.375	5.990	0.036	0.625*
	P	40	165.725	5.862	0.035	
Weight (kg)	F	40	65.375	6.624	0.101	0.659*
	P	40	64.750	5.978	0.092	
BMI	F	40	23.552	1.112	0.047	0.911*
	P	40	23.526	1.005	0.043	
Average Duration (mins)	F	40	108.175	20.528	0.190	0.579*
	P	40	110.200	10.356	0.094	

**Figure 2:**
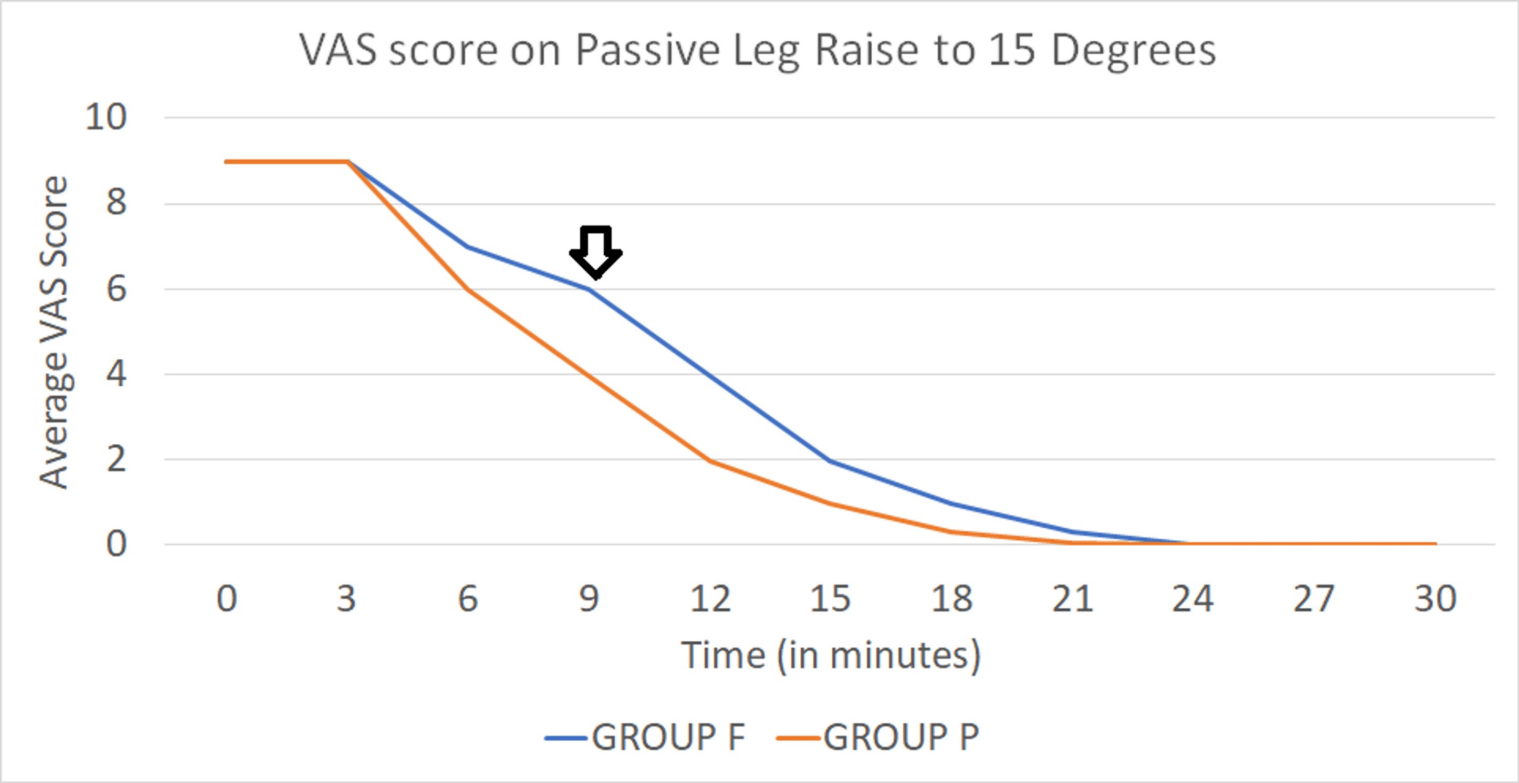
Line diagram comparing the average VAS score between the two groups on PLR to 15 degrees The arrow indicates the change in VAS  score at the ninth minute while comparing the two groups. Group P (orange line) shows an average VAS score of 4 at that point, while Group F(blue line) shows an average VAS score of 6 on PLR to 15 degrees. VAS: Visual analogue scale; PLR: Passive leg raise

The VAS score on leg raised to 15 degrees every three minutes was comparable between the two groups till the ninth minute, after which a difference in the mean VAS scores in Group P (3.975±0.733) than in Group F (VAS score 5.975±0.733) was noted. This was significant on statistical analysis (p < 0.001) and the trend followed till the twenty-first minute, after which the VAS score was 0 in both groups (Figure 2).

**Figure 3:**
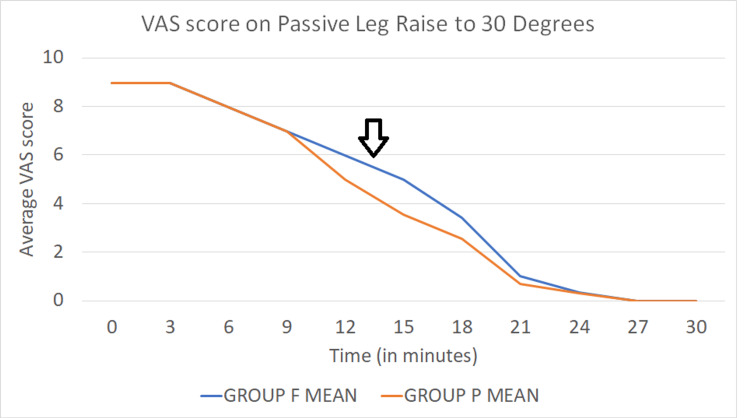
Line diagram comparing the time to PLR to 30 degrees between the two groups The arrow indicates the change in VAS score at the 15th minute while comparing the two groups. Group P (orange line) shows an average VAS score of 4 at that point, while Group F (blue line) shows an average VAS score of 5 on PLR to 30 degrees. VAS: Visual analogue scale; PLR: Passive leg raise

The VAS score on leg raise to 30 degrees every three minutes was comparable between the two groups till 15 minutes, after which there was a significant difference in the mean VAS scores in Group P (VAS score 3.55±0.504) than in Group F (4.975±0.733) (p < 0.001) and the trend followed till the twenty-first minute after which the VAS score was 0 in both the groups (Figure 3).

**Figure 4:**
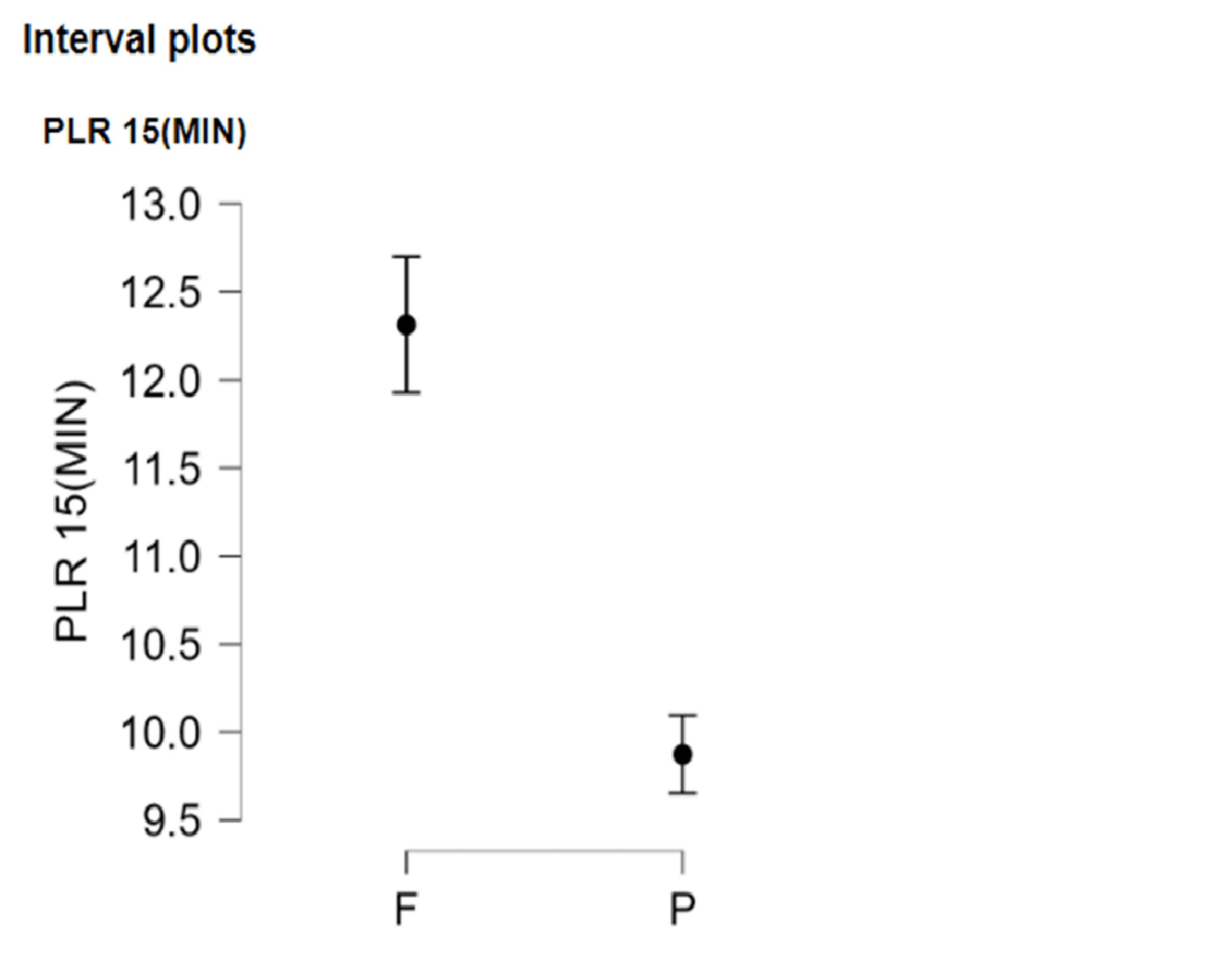
Graph showing time to PLR to 15 degrees at a VAS score < 4 VAS: Visual analogue scale; PLR: Passive leg raise

**Figure 5:**
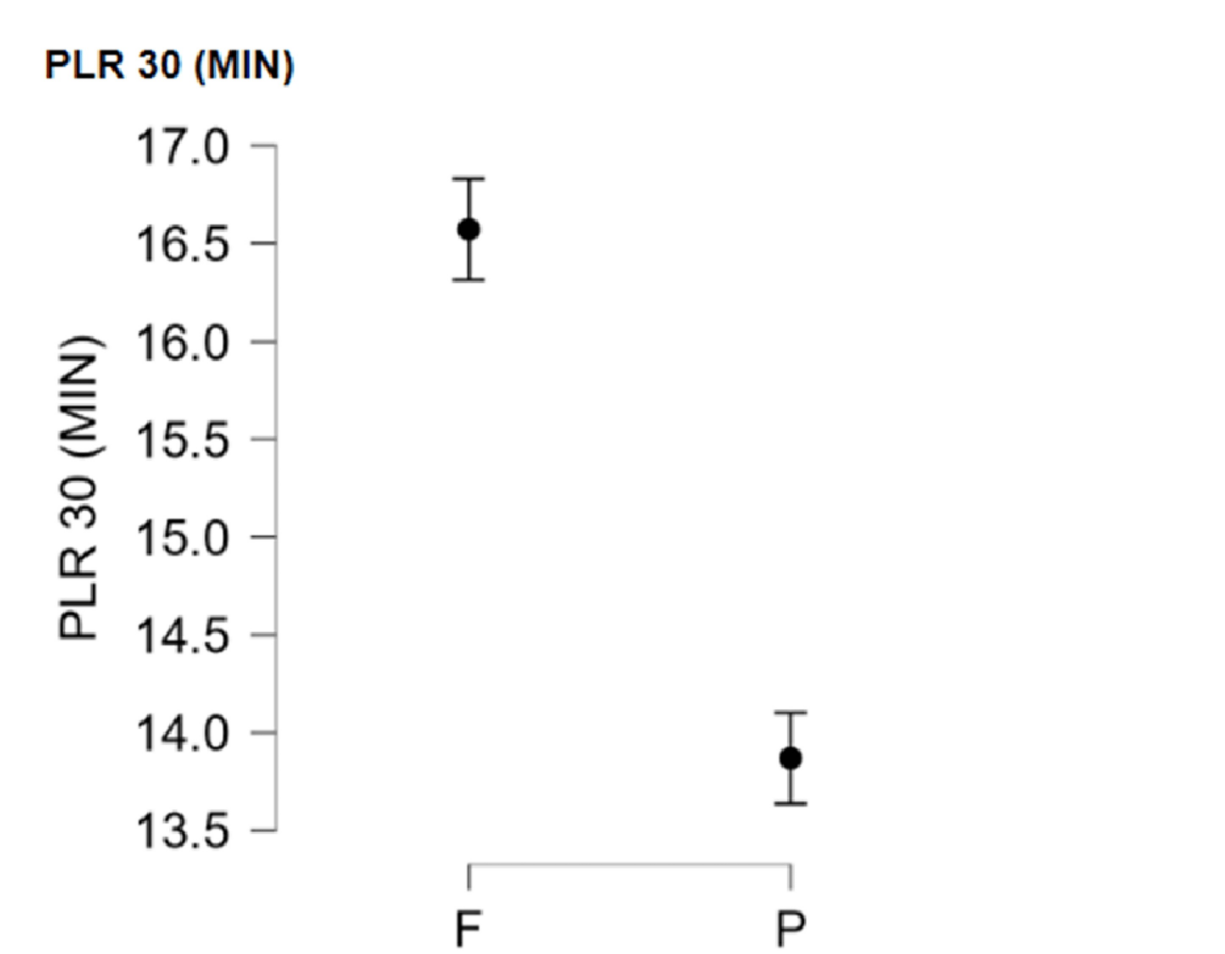
Graph showing time to PLR to 30 degrees at a VAS score < 4 VAS: Visual analogue scale; PLR: Passive leg raise

The time to PLR to 15 degrees was compared between groups. In Group F, the time to a 15-degree PLR was 12.436±0.874 minutes, while in Group P, it was 9.907±0.762 minutes (p < 0.001) (Figure 4). In Group F, the time to perform a 30-degree PLR was longer than Group P (16.573±0.835 vs 13.870±0.749 minutes, respectively, p < 0.001) (Figure 5) (Table 2).

**Table 2:** Comparing PLR at 15 degrees and 30 degrees between the two groups *= p value is insignificant; + = p value is significant. The statistical analysis was done using the independent samples t-test on JASP version 0.18.3. Significance was taken at p < 0.001. PLR: Passive leg raise

Time to PLR	Group F		Group P		Analysis
Time (mins)	Mean	SD	Mean	SD	p-value
PLR 15 degrees	12.436	0.874	9.907	0.762	<0.001 +
PLR 30 degrees	16.573	0.835	13.870	0.749	<0.001 +

Ease of spinal positioning assessed by the means of the patient sitting satisfaction score was compared between the two groups(Table 3) [9]. In Group F, 40 percent of the participants had a score of one (satisfactory), 52.5 percent had a score of two (good) and 7.5 percent had a score of three (optimal). In Group P, 10 percent participants had a score of one (satisfactory), 50 percent had a score of two (good) and 40 percent had a score of three (optimal). The average score in Group F was 1.675±0.616, while in Group P it was 2.300±0.648. This carried significance ( p value < 0.001).

**Table 3:** Comparing quality of spinal positioning through the patient sitting satisfaction score between the two groups *= p value is insignificant , + = p value is significant. The statistical analysis was done using the independent samples t-test on JASP version-0.18.3. Significance was taken at p < 0.001.

Quality of Spinal Positioning (Score)	Group F	Group P	p-value
Mean	1.675	2.300	<0.001 +
SD	0.616	0.648	

The time to first dose of rescue analgesic in the PACU was compared between the two groups, and we found that the average time to the first dose of Injection Paracetamol in Group F was longer than in Group P (7.854±0.726 vs 5.223±0.615 hours, respectively, p < 0.001) (Table 4).

**Table 4:** Comparing the time to first dose of rescue analgesic (Injection Paracetamol) in hours (duration of effective postoperative analgesia) *= p value is insignificant , + = p value is significant. The statistical analysis was done using the independent samples t-test on JASP version-0.18.3. Significance was taken at p < 0.001.

Time to First Dose of Injection Paracetamol (in Hours)	Mean	SD	p-value
Group F	7.854	0.726	<0.001 +
Group P	5.223	0.615	

The average time to first dose of Injection Tramadol (the second rescue analgesic) measured in hours in Group F was longer than Group P (15.957±1.609 hours vs 12.325±1.328 hours, respectively, p < 0.001) (Table 5).

**Table 5:** Comparing the time to first dose of Injection Tramadol (in hours) *= p value is insignificant , + = p value is significant. The statistical analysis was done using the independant-samples t-test on JASP version-0.18.3. Significance was taken at p<0.001.

Time to First Dose of Injection Tramadol (in Hours)	Mean	SD	p-value
Group F	15.975	1.609	< 0.001 +
Group P	12.325	1.328	

The total dose of analgesics required was comparable between the two groups and was statistically insignificant (Table 6).

**Table 6:** Comparing the total amount of rescue analgesics between the two groups *= p value is insignificant , + = p value is significant. The statistical analysis was done using the independent samples t-test on JASP version-0.18.3. Significance was taken at p < 0.001.

Total Amount of Analgesics Consumed	Mean	SD	p-value
Group F	3.825	0.549	0.012 *
Group P	4.150	0.580	

## Discussion

Fractures of the hip contribute to a major public health issue on a global scale and have a substantial impact on the quality of life of the population [10]. Pain management in the perioperative period is essentially multimodal and crucial for early mobilization and discharge rates [11].

During spinal anesthesia, the positioning of patients with intertrochanteric fractures is compromised by extreme pain on flexion at the hip joint. Hence, pre-spinal peripheral nerve blocks play a very crucial role in alleviating such pain in the preoperative period while ensuring prolonged analgesia in the postoperative period [12].

The pain with hip fractures is mainly contributed by the nerve supply of the anterior capsule, which involves contributions from the femoral, obturator, and accessory obturator nerves [13]. The skin over the operable site is supplied by the cutaneous branches of the femoral nerve and the lateral femoral cutaneous nerve of thigh (LFCN). The FICB and the PENG block are the newer blocks that are often administered to patients in this setting for alleviating pain following such hip fractures since they target both the obturator and the femoral nerves of the hip capsule. The FICB has the disadvantage of not being able to target the accessory obturator nerve in most patients. The PENG block, which offers this extra benefit, is therefore proven to be more effective than the former. However, the disadvantage with the PENG block is that the incision site, which is primarily supplied by the LFCN, is not always covered and requires a higher volume of local anesthesia [14]. Here , the FICB seems more superior due to its inclusion of the LFCN [15].

We compared the standard landmark-based FICB with a landmark-based PENG block described by Jadon et al., as described earlier [8]. This is in effort to assess the feasibility of the landmark-based techniques in the resource-poor setting across the country. Studies have compared the ultrasonography-guided FICB and PENG block and have shown the superiority of the PENG blockade for targeting hip fractures for preoperative positioning and have shown benefits for postoperative analgesia as well [16].

The onset of sensory blockade was compared in our study between FICB and PENG block by measuring the time to a comfortable PLR first to 15 degrees, then to 30 degrees. This was followed by assessment of the quality of spinal positioning through the sitting satisfaction score. In the PENG group, the time to PLR to 15 degrees was 9.907±0.762 minutes, while in the FICB group it was 12.436±0.874 minutes, which carried significance (p < 0.001). In the PENG group, the time to passively raise the leg to 30 degrees was 13.870±0.749 minutes, while in the FICB group it was 16.573±0.835 minutes, which was statistically significant (p < 0.001).

The average duration at which the VAS score was < 4 at a 15-degrees followed by 30-degree PLR was attained much faster in the PENG group than the FICB group.

The average patient sitting satisfaction score at 30 minutes was compared between the two groups. In Group P, it was 2.300±0.648, while in Group F, it was 1.675±0.616, which was statistically significant (p < 0.001) . The results show that PENG offers faster onset and better spinal positioning than FICB. This can be attributed to the three nerve targets of the PENG rather than the FICB [17]. Also, the PENG block is administered a little superior to the classically described infra-inguinal FICB, in which the articular branches are more easily missed. Our study shows similar results to the studies done comparing the two blocks using ultrasonography guidance by Jadon et al. and Mosaffa et al., who showed that PENG enabled better positioning and analgesia compared to FICB [9,16,18].

The effect of the two blocks on the duration of postoperative analgesia was compared between the two groups by the time to the first dose of rescue analgesic paracetamol, the time to the second rescue analgesic tramadol, and the total doses of rescue analgesics consumed in the first 24-hour period. The average time to the first dose of injection paracetamol measured in hours in the FICB group was longer than in the PENG group (7.854±0.726 hours vs. 5.223±0.615 hours, respectively, p < 0.001).

Similarly, the average time to the first dose of injection tramadol was longer in the FICB group than the PENG group (15.957±1.609 hours vs. 12.325±1.328 hours, respectively, p < 0.001). This shows that patients administered FICB had a longer pain-free interval than in the PENG group. Our study shows similar results to the cohort study by Sahoo et al. [19].

In our study, the results show that though the PENG block offered superior spinal positioning, the requirement for rescue analgesics was faster. 

A Cochrane review by Farag et al. showed that the PENG block offered superior analgesic benefits that also extended to the postoperative period [20]. A study by Senthil et al. found that in terms of postoperative analgesia, the PENG block had the advantage of motor sparing and superior analgesia [20,21]. Samar et al., in their study, compared the two blocks and showed that the PENG block had the overall advantage of reducing the requirement for analgesics in the perioperative period [22]. However, the results of our study show that while the intraoperative benefits of the PENG block were superior to those of the FICB group, the post-operative analgesia was much longer in the FICB group.

The probable cause can be attributed to the lack of incision site coverage by the PENG block for intertrochanteric fractures, which are situated more postero-laterally in the territory of the LFCN, as described earlier. Though a large volume of local anesthetics is known to alleviate this particular disadvantage of the block, the landmark-guided technique could have been placed at a higher and more medial plane that could easily miss the LFCN. Thus, additional LFCN blockade could be necessary in these patients. This was reported in a study by Liang et al. [23].

The total amount of analgesics consumed was comparable between the two groups.

The side effects that we observed included complaints of nausea, vomiting, headaches, and any episodes of hypotension. We had two participants in the PENG group and one in the FICB group who had complaints of one episode of vomiting. Injections of Ondansetron at a dose of four milligrams were administered to these patients.

Our study did not have any block failure.

Limitations

Postgraduates were not allowed to perform the blocks in our study. Hence, the efficiency of these blocks when performed by relatively novice trainees could not be assessed. We plan to include such trainees in our future studies. Also, this study was limited to intertrochanteric hip fractures. The utility value in other types of fractures must be assessed. The use of nerve stimulator in the PENG block might seem to improve the latter's accuracy. However, the FICB and the PENG block are both inter-fascial, plane blocks and do not require nerve stimulator for identifying the plane. The primary target of utilizing the nerve stimulator in this study was to avoid accidental intraneural injection of some articular branches that might cross the iliopubic eminence (anatomic variation), given that the technique is relatively new. The ability to use PENG block without nerve stimulator guidance could be scope to future studies. Similarly, the utility value of these blocks for pure postoperative analgesia can be prospect to future studies.

## Conclusions

Our study concludes that both the landmark assisted FICB and PENG blocks are efficient and feasible techniques for lower limb nerve blocks. While the landmark-guided PENG block is superior to the landmark-guided FICB for preoperative positioning and analgesia, the duration of postoperative analgesia was relatively shorter in patients with intertrochanteric fractures, when compared to the FICB.
